# Case report: post-stroke interventional BCI rehabilitation in an individual with preexisting sensorineural disability

**DOI:** 10.3389/fneng.2014.00018

**Published:** 2014-06-24

**Authors:** Brittany M. Young, Zack Nigogosyan, Veena A. Nair, Léo M. Walton, Jie Song, Mitchell E. Tyler, Dorothy F. Edwards, Kristin Caldera, Justin A. Sattin, Justin C. Williams, Vivek Prabhakaran

**Affiliations:** ^1^Department of Radiology, University of Wisconsin-MadisonMadison, WI, USA; ^2^Neuroscience Training Program, University of Wisconsin-MadisonMadison, WI, USA; ^3^Medical Scientist Training Program, University of Wisconsin-MadisonMadison, WI, USA; ^4^Department of Biomedical Engineering, University of Wisconsin-MadisonMadison, WI, USA; ^5^Departments of Kinesiology and Medicine, University of Wisconsin-MadisonMadison, WI, USA; ^6^Department of Orthopedics and Rehabilitation, University of Wisconsin-MadisonMadison, WI, USA; ^7^Department of Neurology, University of Wisconsin-MadisonMadison, WI, USA; ^8^Department of Neurosurgery, University of Wisconsin-MadisonMadison, WI, USA; ^9^Departments of Psychology and Psychiatry, University of Wisconsin-MadisonMadison, WI, USA

**Keywords:** stroke rehabilitation, brain-computer interface, case study, disability, BCI therapy, UE motor rehabilitation, BCI-FES-TDU

## Abstract

Therapies involving new technologies such as brain-computer interfaces (BCI) are being studied to determine their potential for interventional rehabilitation after acute events such as stroke produce lasting impairments. While studies have examined the use of BCI devices by individuals with disabilities, many such devices are intended to address a specific limitation and have been studied when this limitation or disability is present in isolation. Little is known about the therapeutic potential of these devices for individuals with multiple disabilities with an acquired impairment overlaid on a secondary long-standing disability. We describe a case in which a male patient with congenital deafness suffered a right pontine ischemic stroke, resulting in persistent weakness of his left hand and arm. This patient volunteer completed four baseline assessments beginning at 4 months after stroke onset and subsequently underwent 6 weeks of interventional rehabilitation therapy using a closed-loop neurofeedback BCI device with visual, functional electrical stimulation, and tongue stimulation feedback modalities. Additional assessments were conducted at the midpoint of therapy, upon completion of therapy, and 1 month after completing all BCI therapy. Anatomical and functional MRI scans were obtained at each assessment, along with behavioral measures including the Stroke Impact Scale (SIS) and the Action Research Arm Test (ARAT). Clinically significant improvements in behavioral measures were noted over the course of BCI therapy, with more than 10 point gains in both the ARAT scores and scores for the SIS hand function domain. Neuroimaging during finger tapping of the impaired hand also showed changes in brain activation patterns associated with BCI therapy. This case study demonstrates the potential for individuals who have preexisting disability or possible atypical brain organization to learn to use a BCI system that may confer some rehabilitative benefit.

## Introduction

Each year approximately 795,000 individuals experience a new stroke in the United States alone (Go et al., 2014), with up to 50% of stroke survivors suffering from some form of persistent neurological impairment (Kelly-Hayes et al., 2003). Brain-computer interface (BCI) technology is being incorporated into an emerging class of devices designed to facilitate the rehabilitation of individuals with persistent motor impairments after stroke (Buch et al., 2008; Daly et al., 2009; Broetz et al., 2010; Prasad et al., 2010; Caria et al., 2011; Shindo et al., 2011; Liu et al., 2012; Takahashi et al., 2012). These BCI systems detect the user's neural signals, translate these signals into action, and provide real time feedback using various feedback modalities, including visual displays (Prasad et al., 2010), robot-assisted movement (Buch et al., 2008; Broetz et al., 2010; Caria et al., 2011; Ramos-Murguialday et al., 2013; Varkuti et al., 2013), functional electrical stimulation (FES) (Daly et al., 2009; Takahashi et al., 2012), and tongue stimulation (TDU) (Wilson et al., 2012).

Currently, research into the feasibility and efficacy of such BCI devices for applications in stroke rehabilitation focuses largely on individuals whose neurological impairments have been acquired as a direct result of their stroke event. However, there is some precedent in the feasibility of individuals with disabilities from other etiologies successfully learning to use machine interfaces (Sampaio et al., 2001; Bach-Y-Rita, 2004) and BCI devices. For example, it has been shown that blind individuals can learn to use BCI systems through the use of electrotactile feedback, performing comparably to sighted individuals using visual feedback (Wilson et al., 2012). BCI systems may also be implemented as a means of replacement or augmentative function for individuals who have locked-in syndrome (Kaufmann et al., 2013; Oken et al., 2013; Lugo et al., 2014). However, less is known about the behavioral and neuroimaging changes that may be induced by such BCI systems when used for rehabilitation in individuals with neurological impairments and disabilities prior to stroke.

Approximately 30% of non-institutionalized individuals in the United States over the age of 65 report having a visual, hearing, or cognitive disability (Institute, 2013). Furthermore, 6–13% of individuals in this same age group suffer a stroke each year (Go et al., 2014), resulting in a need for rehabilitative therapies that can be made available to stroke survivors who may have a history of disability prior to stroke. We report the use of a BCI system with coordinated visual, FES, and TDU feedback modalities designed to improve the rehabilitation of upper extremity motor impairment by a subacute stroke subject with a preexisting sensorineural disability (congenital deafness) and a history of depression prior to stroke.

## Materials and methods

### Case description and participant recruitment

**Figure 1:**
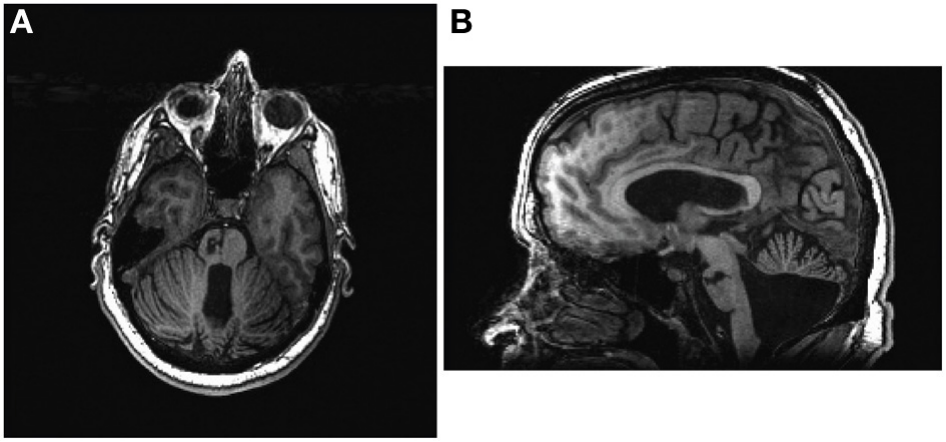
**The participant's lesion can be seen in the right pons in both axial (A) and sagittal (B) views**. Axial image is presented in radiological conventions.

**Figure 2:**
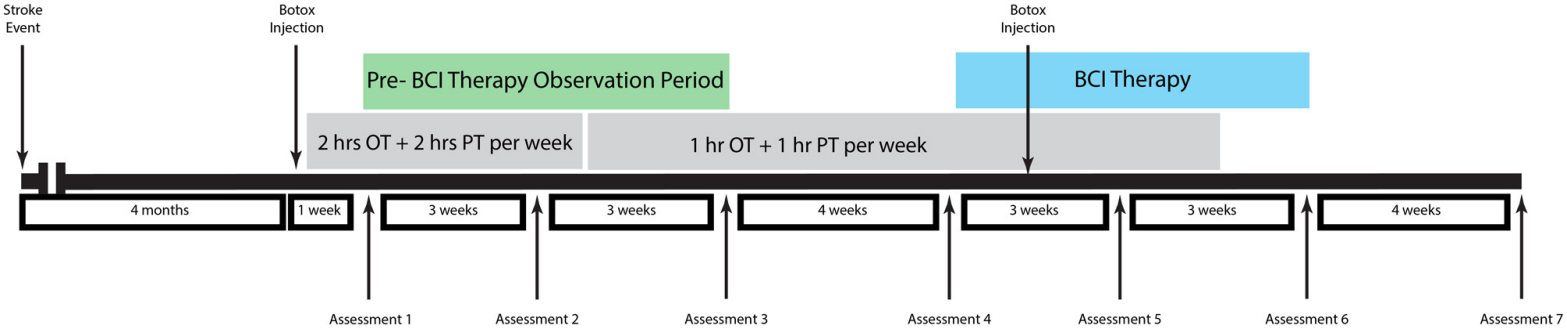
**Therapy and assessment schedule**.

The participant was a 48-year old male stroke patient whose pre-stroke medical history was significant for deafness due to congenital rubella infection, along with depression and diabetes mellitus. Prior to stroke, the participant had been left-handed with a score of -78 on the Edinburgh Handedness Inventory (Oldfield, 1971) and communicated in ASL using both hands with his left hand as the dominant signing hand. He suffered an ischemic stroke in the right pons (Figure 1), which resulted in persistent left-sided hemiparesis. The participant began assessments in this study approximately 4 months after stroke onset. At the time of study enrollment, the participant's medications included sertraline 50 mg daily and metformin 1000 mg daily, which were sufficient to keep his depression and diabetes respectively under control throughout the study period. Other medications included aspirin 325 mg daily, simvastatin 20 mg daily, and Lisinopril 5 mg daily. The subject also received botulinum toxin injections just prior to the beginning of study participation as well as during study participation (Figure 2) and took oral baclofen 10 mg three times daily to reduce spasticity. At the time of the study, the participant was able to read and understand written English, although his ability to write with his non-dominant right hand was slow and clumsy. This research was approved by the local Institutional Review Board. The participant provided written informed consent.

### Therapy and assessment schedule

Neuroimaging and behavioral measures were assessed at three time points prior to the administration of any BCI therapy during a pre-therapy observational period. BCI therapy consisted of 13 2-h interventional therapy sessions using the BCI-FES-TDU system over the course of 6 weeks with no more than three interventional therapy sessions per week.

Behavioral and neuroimaging assessments were then repeated immediately prior to the beginning of BCI therapy, mid-therapy after 3 weeks of intervention sessions, upon the completion of all therapy sessions, and 1 month after the conclusion of all therapy sessions. The subject also received 1–2 h per week of additional occupational therapy and physical therapy during part of the study period administered independently from therapy sessions using the BCI-FES-TDU system. An assessment and treatment timeline for this patient is summarized in Figure 2.

### Behavioral outcome measures

Both objective and subjective behavioral measures were assessed at each assessment time point. Objective measures included the Action Research Arm Test (ARAT) (Carroll, 1965; Lyle, 1981; Lang et al., 2006) and grip strength averaged over three attempts as measured by dynamometry. Subjective measures included transformed scores for each subdomain of the Stroke Impact Scale Version 3.0 (SIS) (Duncan et al., 1999; Carod-Artal et al., 2008), the Motor Activity Log (MAL) (Uswatte et al., 2005, 2006), the Wong-Baker pain scale (Garra et al., 2013), and the Center for Epidemiologic Studies Depression Scale (CES-D) (Radloff, 1977; Eaton et al., 2004; Carleton et al., 2013). The Modified Ashworth Scale (Bohannon and Smith, 1987) was also used to measure spasticity in the wrist and fingers of the impaired left side at each assessment.

### MRI acquisition and processing

All MR images were acquired on a 3 Tesla GE MR750 scanner equipped with high-speed gradients (Sigma GE Healthcare, Milwaukee Wisconsin) using an 8-channel head coil. Padding was used to minimize head movement. Functional scans were run using a T2^*^-weighted gradient-echo echo planar imaging (EPI) pulse sequence sensitive to BOLD contrast. Technical parameters used to acquire EPI scans were: field of view 224 mm, matrix 64 × 64, TR 2600 ms, TE 22 ms, flip angle 60°, and 40 axial plane slices of 3.5 mm thickness with 3.5 mm spacing between slices. During each fMRI scan, 70 sequential whole-brain acquisitions were recorded. During EPI scans, the subject was cued to alternate between finger tapping with the left hand and rest in blocks of 20 s. In order to cue the participant to alternate between these 20 s blocks, a member of the research team tapped the subject lightly on the arm at the beginning of each 20 s block. This type of cueing was chosen because the participant's vision, while corrected to normal outside of the scanner with glasses, could not be sufficiently corrected within the scanner to allow the participant to read cues projected on a screen. Due to insufficient grip strength and impaired motor control, the subject was unable to use a button box during finger tapping. Instead, the researcher in the scan room who cued the subject also observed the subject's hand to ensure that visible attempted finger tapping or rest occurred as appropriate during each block. Up to three T1-weighted high-resolution anatomical images were also obtained during each scanning session using a BRAVO FSPGR pulse sequence. Technical parameters used to acquire anatomical scans were: field of view 256 mm, matrix 256 × 256, TR 8.16 ms, TE 3.18 ms, flip angle 12°, and 156 axial plane slices of 1 mm thickness with 1 mm spacing between slices.

All pre- and post-processing of MRI data was performed using the AFNI software package (Cox, 1996). The first four volumes of each functional sequence were discarded to allow for signal stabilization. EPI data sets were motion corrected and then spatially smoothed at 6 mm with a full width at half maximum Gaussian kernel. Each voxel timeseries was scaled to a mean of 100, and AFNI's 3dDeconvolve was then used to perform a voxel-wise regression analysis with six motion parameters regressed out. This analysis yielded a voxel-wise t-statistic. EPI data sets were visually inspected for alignment with anatomical T1 datasets, with the best available dataset for each scan session used for alignment. All scan sessions had at least one anatomical T1 dataset with adequate alignment upon visual inspection. Activation maps were then transformed into Talairach space and 3dcalc used to create difference maps across the pre-therapy control and BCI therapy periods. These difference maps were then cluster corrected for multiple comparisons with a minimum cluster size 229 voxels, as calculated using AFNI's 3dClustSim, and thresholded at *t* = 2.674 (*p* < 0.01).

### Interventional therapy description

Brain activity was recorded using a 16-channel EEG cap (g.GAMMA cap, Cortech Solutions) and amplifier (Guger Technologies) and processed using BCI2000 software (Schalk et al., 2004) version 2 with in-house modifications to allow for administration of additional tongue stimulation (TDU 01.30, Wicab Inc.) and functional electrical stimulation (LG-7500, LGMedSupply; Arduino 1.0.4).

At the start of each interventional therapy session, the participant was asked to rank how motivated he was to continue participating in this study on a scale of 1–10, with 1 being not motivated and 10 being extremely motivated.

Each session of interventional therapy using the brain-computer interface device then consisted of three stages. The participant first performed open-loop attempted movement of each hand with no performance feedback alternating with periods of rest. Each of these conditions (i.e., attempted movement of the right (or left) hand, or rest) was prompted at least 10 times for 4 s per prompt. The movements used during attempted movement were repeated opening and closing of each hand, which the subject was able to perform although movements using his impaired left hand were noticeably slower and more limited (see Video S1).

Attempted movements were used during both the open-loop screening and subsequent closed-loop feedback tasks in order to allow for the mental processes trained during BCI therapy to be as similar as possible to those needed when attempting functional movements beyond the laboratory environment. Motor imagery is a popular means of controlling BCI devices and is found more commonly in the BCI literature than attempted movement. However, this is due largely to the history of the field and is less reflective of an inherent limitation of BCI devices. Many BCI systems were designed with an augmentative purpose in mind, intended to allow for communication in individuals with permanent or progressive motor impairments (Wolpaw et al., 2000). Early BCI systems were also developed using individuals free of motor impairments (Leuthardt et al., 2004) or in individuals whose motor impairments were not targeted by the paradigms used (Wolpaw and McFarland, 2004). In such studies, motor imagery represented a means of establishing control of a device independent of the need for actual movements that a later user with motor impairments relevant to the trained task may not be able to perform. The largely augmentative aims of many BCI devices and the testing of early BCI systems on individuals who were not necessarily affected by the motor impairments that these systems were designed to address contributed to the preferential use of motor imagery as a way to control BCI devices by allowing for early machines to be adapted for the benefit of the largest number of potential users where large variations may exist in the degree of individual motor impairment.

Motor imagery continues to be a good option for the control of a BCI device and has been incorporated into a number of rehabilitative (Buch et al., 2008; Daly et al., 2009; Broetz et al., 2010; Prasad et al., 2010; Caria et al., 2011; Shindo et al., 2011; Varkuti et al., 2013) and augmentative (Kubler et al., 2005) systems. However, while the goals and populations studied during the development of early BCI devices helped to establish motor imagery as a standard method of control, this precedent does not preclude the use of other mental tasks as potential options for the control of BCI devices (Felton et al., 2007). Furthermore, while the original definition of BCI devices emphasized that these devices could be controlled using motor imagery and mental tasks that do not require the production of actual movement, these definitions did not exclude the possibility that mental tasks accompanied by actual movements could be used for BCI control (Wolpaw et al., 2000). Some newer BCI devices have also begun incorporating attempted movement into their protocols, particularly when the system is intended to serve a rehabilitative purpose (Daly et al., 2009; Prasad et al., 2010; Takahashi et al., 2012; Ramos-Murguialday et al., 2013; Mukaino et al., 2014).

The goal for therapy with the BCI system used in this study was purely rehabilitative rather than augmentative, with stimulus from the BCI device functioning as both a feedback modality as well as a form of assistive support for the production of actual movements in order help to strengthen or reestablish a lost functional capacity. Therefore, actual movements were used to identify appropriate control signals for neural feedback, and the subject was taught to use attempted movements of each hand to control the BCI device. This approach maximizes the similarities between mental tasks trained during BCI therapy and those produced when attempting functional movement in the real world, so that gains made with the device might persist beyond the therapy period. While the device used in this study is controlled by attempted movement rather than imagined movement, we believe that it may still be classified as a BCI system because it allows for neural activity patterns to be detected and translated into a computer-generated feedback response in real time.

Data from open-loop trials was analyzed offline to determine appropriate EEG-based control features for subsequent closed-loop tasks. This initial calibration task and its application to control feedback during later stages of the therapy session is based on previously described processes (Wilson et al., 2009). In summary, the BCI2000 Offline Analysis tool was used to determine the channels for in which the largest r-squared values were found within the frequency ranges for the Mu and Beta rhythms for each condition of attempted movement using either the right or left hand. These channels and the specific frequency bins for which the largest r-squared values were identified were then used as control signals for the closed-loop neurofeedback task. For this participant, control signals were based on the desynchronization of Mu and Beta rhythms detected over the sensorimotor cortex by electrodes placed on the scalp in positions C3, CP3, Cz, C4, and CP4 using the international 10–20 system.

The next stage of the intervention involved a closed-loop condition in which the participant was presented with real time visual feedback to allow him to learn how to modulate cortical activity. Visual feedback was presented in the context of a game in which a target would appear on the left or right side of the screen and the participant was instructed to move a cursor from the center of the screen to the target using attempted hand movement. In this closed-loop condition, attempted movement consisted of either repeated opening and closing of each hand similar to that used for open-loop screening or repeated wrist extension. These actions were used because the participant expressed the desire to improve his ability to open his hand and extend his wrist over other types of movements. The feedback component of this visual display was the lateral movement of the cursor toward or away from the on-screen target, which was controlled in real time by the participant's EEG signals. Attempted hand movements were used to control cursor movement during all trials in this stage as well as in all trials of all subsequent stages. Cortical activity related to attempted movement of the right (left) hand as detected by EEG was translated into rightward (leftward) movement of the on-screen cursor. Each run consisted of 8-12 trials, with each trial randomly presenting one of four possible targets. The participant was given the goal of completing at least 10 runs for this stage as well as during the final stage.

The third stage of the intervention session was similar to the second, using the same game play paradigm with the incorporation of additional feedback in the form of TS and FES to muscles of the impaired left arm to assist with the impaired attempted movement. FES electrodes were applied over the extensor carpi radialus brevis and extensor digitorum muscles in the left forearm to assist with wrist and finger extension. These muscles were chosen for stimulation after the participant reported having more baseline volitional control over active flexion of his wrist and fingers than over wrist and finger extension. TS feedback paralleled visual feedback and provided continuous electrotactile stimulation of the tongue during each trial. Furthermore, TS has been shown to provide sufficient feedback to enable a subject to use a similar BCI device with TS alone in the absence of visual or other tactile feedback (Wilson et al., 2012) and has also been implicated in priming neuromodulation (Wildenberg et al., 2010). TS with this device was organized in a grid that delivers electrical stimulus representing the positions of the on-screen cursor and target onto the tongue. FES was triggered when cortical activity related to attempted movement of the impaired limb was detected by EEG and the participant had been cued to attempt movement of the impaired hand (i.e., when the target was on the left). Thus, since both cursor movement and FES were controlled by the same set of EEG signals, FES was only applied when the cursor moved correctly toward the target on the impaired left side of the body. This triggering of the FES was significant in that it ensured that only consistent, desired patterns of brain activity associated with attempted movement of the impaired left hand were rewarded with feedback from the FES device.

**Figure 3:**
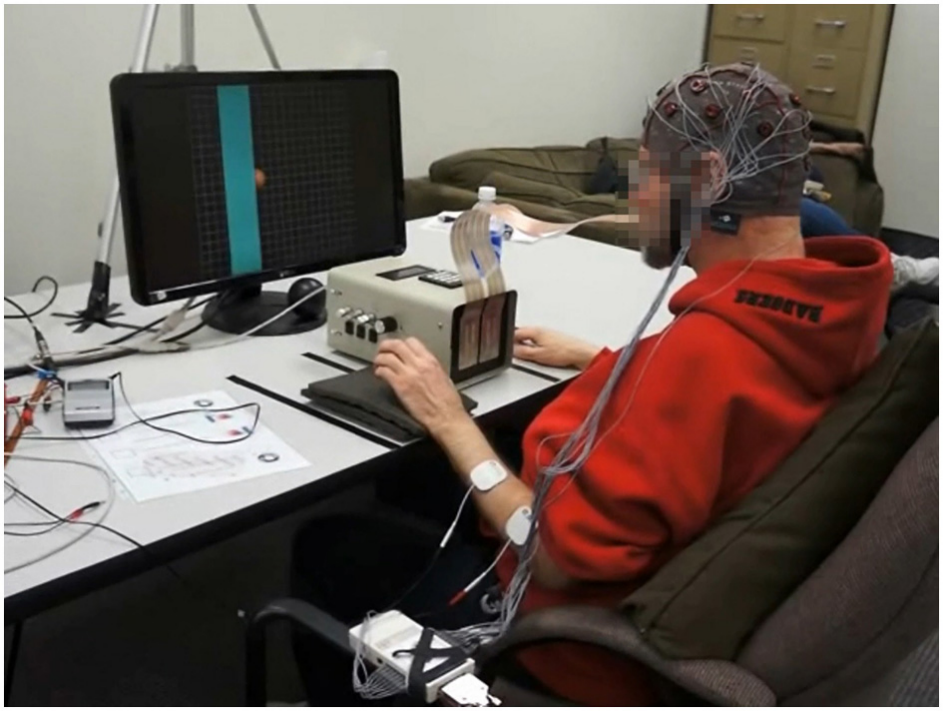
**Study participant using the BCI-FES-TDU system**.

The participant was offered the opportunity to take a short rest break after each stage of the therapy session and was also allowed to take additional short breaks upon request. A picture of game play using all feedback modalities with the BCI-FES-TDU device is provided in Figure 3.

## Results

### Characterization of baseline impairments

At 4 months after stroke before the administration of any therapy using the brain-computer interface system, the participant had left-sided hemiparesis, which manifested as gait disturbances (the participant walked with assistance of a four-pronged cane) as well as spasticity and weakness in the left shoulder, wrist, elbow, and fingers. At 4 months post-stroke, he signed using his right hand as the dominant hand with little to no use of his left hand while signing, whereas he had been strongly left-handed and used his left hand as his dominant signing hand prior to stroke. A baseline ARAT assessment of each upper extremity revealed a perfect score of 57 with no deficits in the unimpaired right hand in contrast to significant deficits and a total score of 26 for the impaired left hand. The breakdown of these scores by each subdomain of the ARAT is presented in Table 1. As can be seen in Table 1, the most severely impaired aspects of the participant's upper extremity motor deficit were in the domains of grip and pinch. A baseline measurement of grip strength showed that the subject was unable to produce any measureable grip strength on a standard dynamometer, which is consistent with the significant deficits evidenced in the subject's Grip subscore from the ARAT of his left upper extremity administered on the same day.

**Table 1:** **Scores for each subdomain of the ARAT administered at the participant's first visit baseline assessment for the impaired and unimpaired upper extremities**.

**ARAT subdomain**	**Score for impaired left UE**	**Score for unimpaired right UE**
Grasp	17	18
Grip	4	12
Pinch	2	18
Gross Movement	3	9

ARAT, Action Research Arm Test; UE, Upper Extremity.

### Participant compliance and clinical observations

The participant used the closed-loop neurofeedback BCI device successfully and tolerated the entire course of therapy and assessments without problems, ranking his motivation to continue participating in the study at a 7 or 8 out of 10 throughout the 6 weeks of therapy and answering “No” when asked at the end of all BCI therapy if he had experienced any side effects. It was also noted that, while all ASL communication observed prior to BCI therapy administration used only the participant's right hand, by the end of the BCI therapy period he had begun using his impaired left hand in a limited fashion to assist in the formation of two-handed signs.

### BCI results

**Figure 4:**
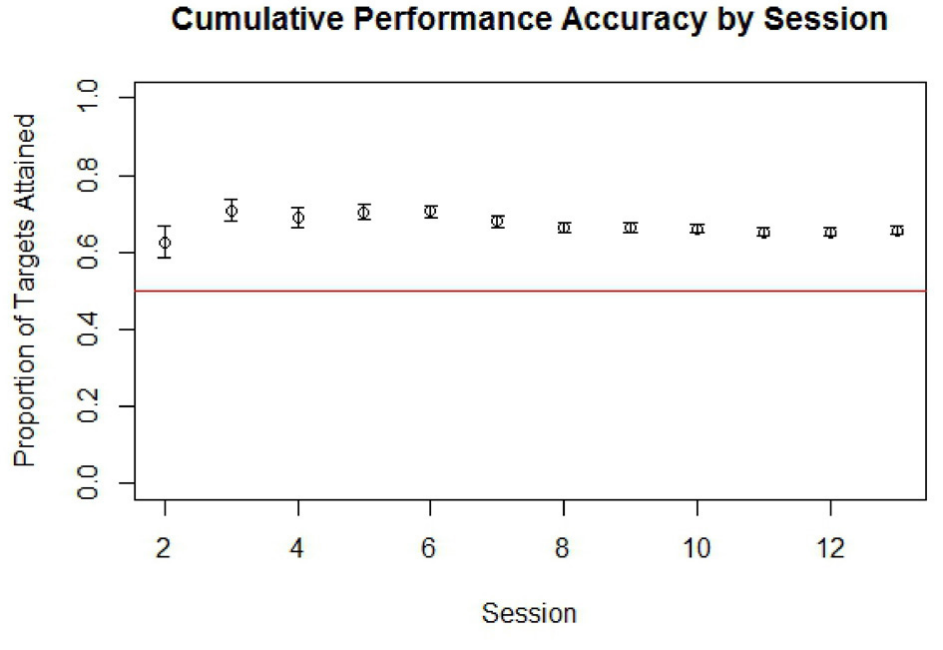
**Cumulative performance accuracy in targets attained by run for all completed non-adaptive runs of BCI cursor task game play grouped by session**. The red line in is drawn at 0.5, which represents the level at which half of targets presented within a run would be attained by chance. Error bars represent the standard error.

The participant was able to learn to use the BCI device, performing with accuracy consistently greater than chance over the course of the therapy period. Cumulative performance accuracy calculated using all completed non-adaptive runs over subsequent sessions is shown in Figure 4. An analysis of trials by target location also showed that the subject achieved over 70% accuracy when presented with left-sided targets and over 60% accuracy when presented with right-sided targets when averaged over all sessions.

Although 70% accuracy is sometimes viewed in more traditional studies as a minimum criteria for establishing control of a BCI system, this convention was initially established in the context of a Language Support Program where an accuracy of 70% or more was needed in order to make communication possible (Kubler et al., 2001, 2005). In contrast, the BCI system used in this study was not designed as an augmentative communication system and therefore may not require such a high degree of overall performance accuracy in order to establish BCI control. Furthermore, as game play parameters were dynamically adjusted to make the game more difficult as the participant achieved 70% accuracy at a given level or difficulty, his overall accuracy was not necessarily expected to be at or above 70%. A binomial also showed the participant's performance accuracy during non-adaptive runs to be significantly better than chance (*p* < 0.0001), indicating that cursor movement was indeed non-random and controlled to some degree by the participant.

### Behavioral measures

**Figure 5:**
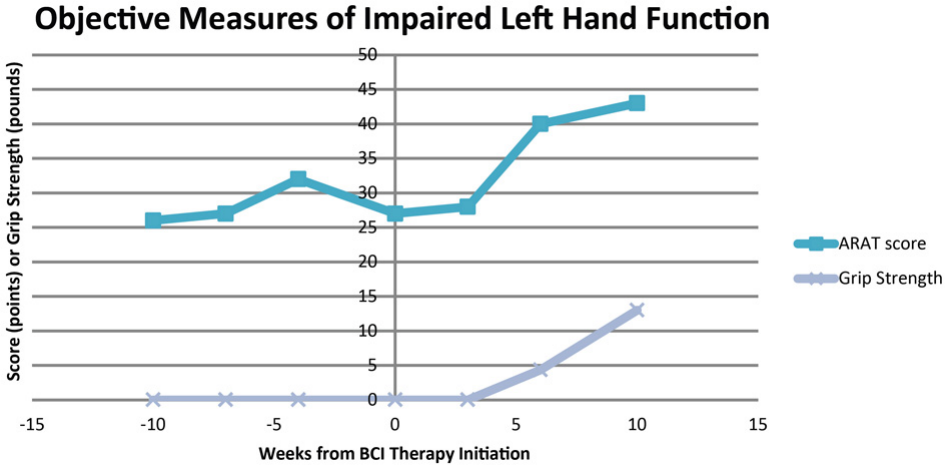
**Scores for ARAT performance and average grip strength of the impaired left hand**. ARAT, Action Research Arm Test.

The participant showed improvement in both ARAT performance using his impaired left hand and in grip strength of his impaired hand with the administration of BCI therapy. These improvements in ARAT performance with BCI therapy administration were greater than improvements observed during the pre-therapy assessments. Specifically, ARAT scores using the impaired left hand varied from 26 to 32 during the pre-therapy observation period and increased from a score of 27 just before BCI therapy to 40 upon the completion of all therapy and 43 1 month after the cessation of BCI therapy. Similarly, no improvements were appreciated in grip strength measurements until after the completion of BCI therapy, registering an average of 4.33 pounds after the completion of all BCI therapy and 13 pounds 1 month later. The participant's performance on the ARAT and his average measured grip strength using his impaired left hand evaluated at each assessment are summarized in Figure 5.

**Figure 6:**
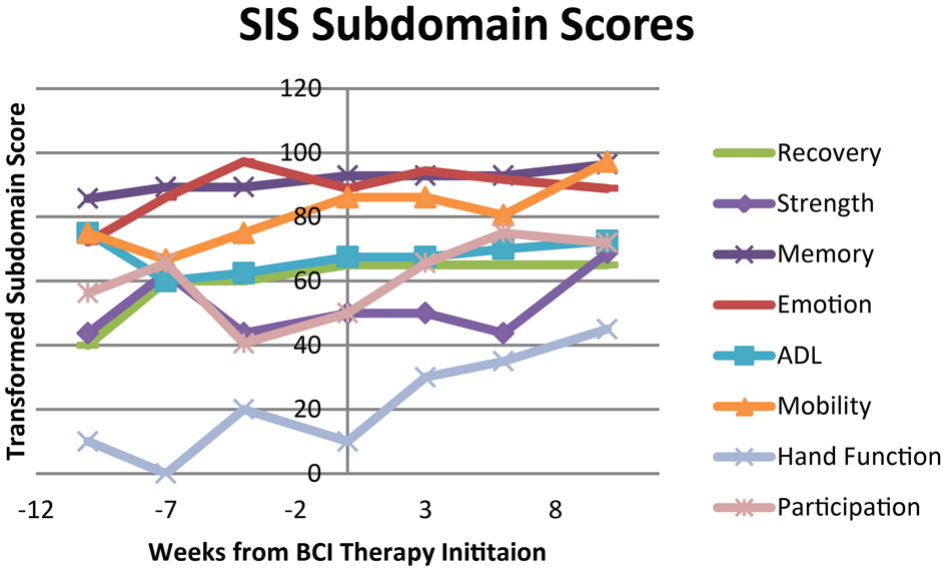
**Transformed scores for each of the subdomains on the Stroke Impact Scale (SIS)**. ADL, Activities of Daily Living.

**Figure 7:**
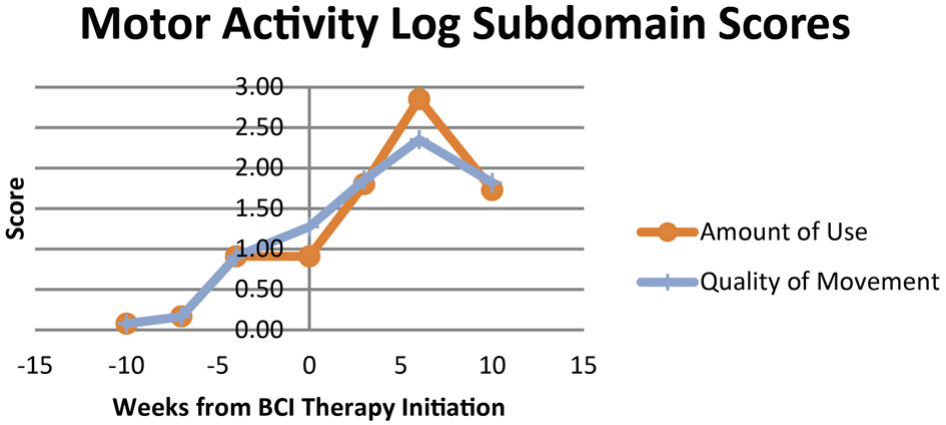
**Scores at each assessment for each domain of the motor activity log**.

The subjective behavioral measures evaluated at each assessment are shown in Figure 6, demonstrating scores for each subdomain of the SIS, and in Figure 7, showing results for the each component of the MAL. Subdomain scores for the SIS remained steady or improved over the course of the study period. Some of the greatest gains were observed in the SIS subdomains for hand function and participation, which demonstrated greater gains during and after the period of BCI therapy administration than during initial pre-therapy observation period.

Scores for both the Amount of Use and Quality of Movement aspects of the MAL increased from first measurement during the observational assessment period to the measurement assessed immediately before the beginning of BCI therapy administration. These scores then both continued to increase during the period of BCI therapy administration, peaking at the assessment immediately after the end of BCI therapy. Scores in each of these domains did demonstrate some decline when reassessed 1 month later relative to the assessment immediately after BCI therapy, but even with this observed decline remained higher than scores for all pre-therapy assessments.

Scores on the CES were consistently below 16 points throughout the study period, which is considered to be the threshold for depression using this screening tool (Radloff, 1977; Eaton et al., 2004). Pain scores also decreased during the study period, reaching zero (i.e., no pain) by the pre-therapy assessment and remaining at this level throughout the remainder of the study.

**Figure 8:**
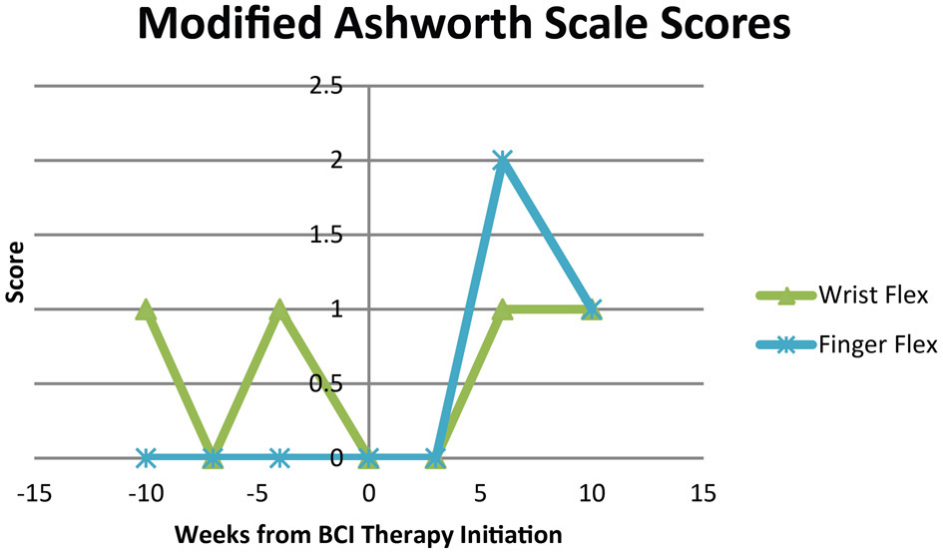
**Modified Ashworth Scale scores for flexion of the wrist and fingers at each assessment**.

Modified Ashworth Scale scores for spasticity during extension of the wrist and fingers were 0 at all time points. Modified Ashworth Scale scores for wrist flexion and finger flexion are shown in Figure 8. Scores were 0 or 1 for wrist flexion throughout the study period and varied from 0 to 2 for finger flexion over the course of study participation.

### *fMRI* results

**Figure 9:**
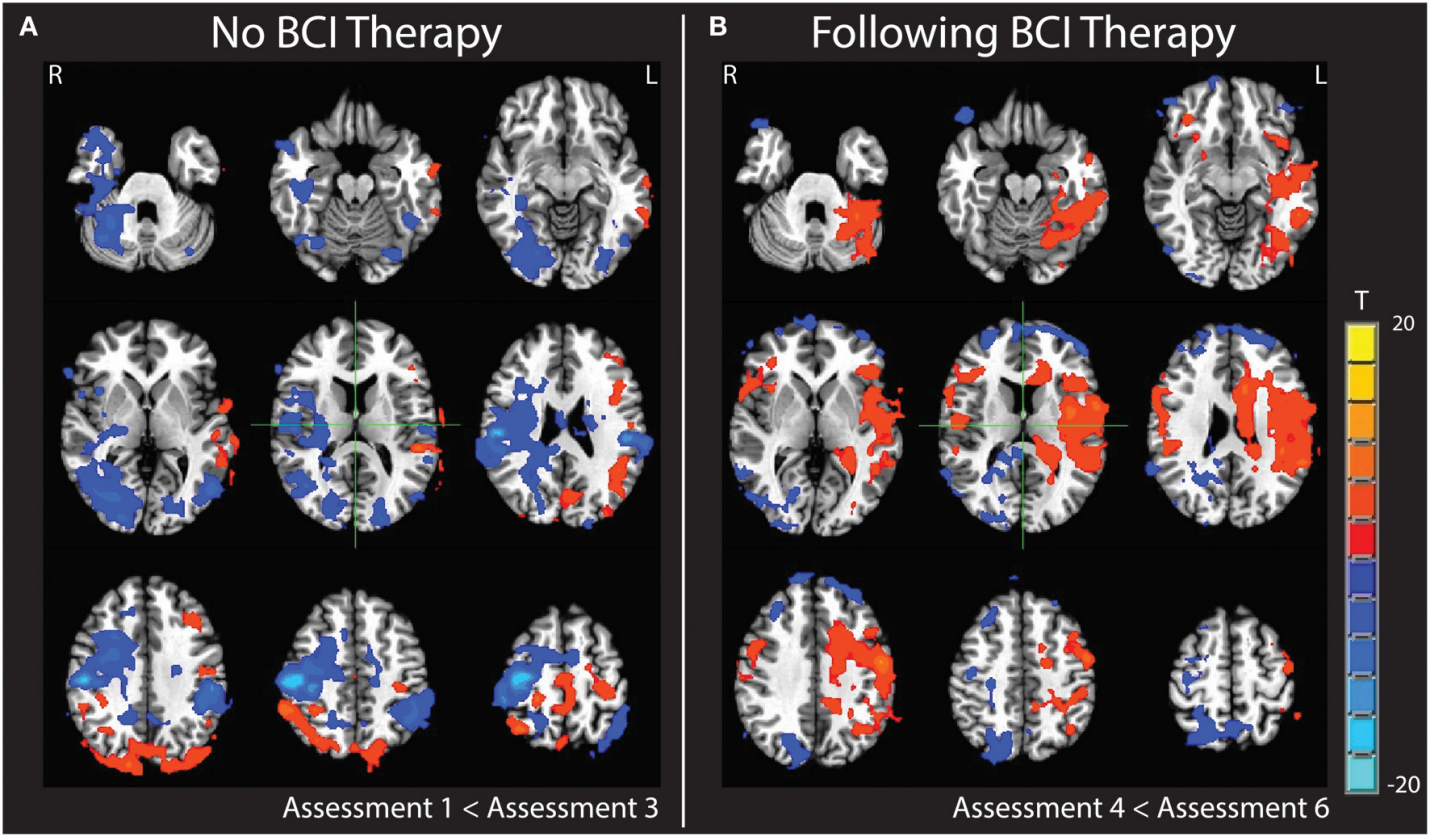
**Subtraction maps demonstrating differences in brain activation during finger tapping of the impaired hand**. Maps compare activation patterns at week 6 of observational control (assessment 3) minus initial baseline activation (assessment 1) **(A)** and changes observed comparing activation post-therapy (assessment 6) minus pre-therapy (assessment 4) **(B)**.

Changes were noted in brain activation elicited during finger tapping of the impaired left hand with the administration of BCI therapy. These changes differed from changes observed when no BCI therapy was given. Specifically, a comparison of baseline activation with activation 6 weeks later during a period in which no BCI therapy was administered showed areas of decreased activation throughout the right hemisphere and right cerebellum. In contrast, a comparison of brain activation during the same in-scanner task before and after the period during which BCI therapy was being administered showed increases in activation throughout the left hemisphere and left cerebellum. These changes are demonstrated in Figure 9. Cluster sizes, direction of activation change, and the Talairach coordinates and location for the focus of each significant cluster are provided in Tables 2, 3.

**Table 2:** **Changes in brain activation during finger tapping of the impaired left hand from assessment 1 to assessment 3 during which no BCI therapy was administered**.

**Cluster**	***X***	***Y***	***Z***	**Region of cluster focus**	**Size (voxels)**	**Increase or decrease in activation**
1	−49.5	22.5	41.5	Right postcentral gyrus	10,662	Decrease
2	49.5	64.5	2.5	Left inferior temporal gyrus	789	Decrease
3	31.5	31.5	50.5	Left postcentral gyrus	476	Increase
4	46.5	31.5	14.5	Left superior temporal gyrus	378	Increase
5	46.5	40.5	−45.5	Left cerebellum	369	Increase
6	43.5	−19.5	20.5	Left middle frontal gyrus	259	Increase

Coordinates provided are in Talairach space.

**Table 3:** **Changes in brain activation during finger tapping of the impaired left hand from assessment 4 to assessment 6 during which BCI therapy was administered**.

**Cluster**	***X***	***Y***	***Z***	**Region of cluster focus**	**Size (voxels)**	**Increase or decrease in activation**
1	49.5	7.5	35.5	Left precentral gyrus	6582	Increase
2	−22.5	52.5	56.5	Right superior parietal lobule	2342	Decrease
3	46.5	−43.5	11.5	Left middle frontal gyrus	1093	Decrease
4	−34.5	−34.5	−3.5	Right middle frontal gyrus	923	Increase
5	−43.5	−37.5	−12.5	Right middle frontal gyrus	230	Decrease

Coordinates provided are in Talairach space.

## Discussion and clinical implications

The results of this study show that it is possible for a stroke survivor with preexisting neurological impairments, in this case sensorineural deafness, to effectively use a BCI device during interventional rehabilitation therapy. The participant's ability to use the BCI device without issue is supported by his consistent performance above chance (Figure 4) along with his self report indicating that he experienced no side effects during the therapy period. Furthermore, the participant achieved gains in both objective and subjective behavioral measures during the time period concurrent with the administration of BCI therapy that were greater than any gains observed during the pre-therapy observation period during which no BCI therapy was administered. These functional gains were accompanied by changes in brain activation observed during attempted finger tapping of the impaired hand, which were again observed with the administration of BCI therapy but not during the pre-therapy observation period.

To our knowledge, this is the first reported case of a deaf individual learning to use a BCI device for the purpose of motor rehabilitation. There have been a number of studies examining the feasibility of adapting BCI devices for use by individuals with visual impairments (Guo et al., 2010; Wilson et al., 2012; Lim et al., 2013; McCreadie et al., 2013) and numerous studies on the ability of individuals with conditions such as amyotrophic lateral sclerosis to control BCI devices (Kubler et al., 2005; Bai et al., 2010). However, in the majority of such cases the use of the BCI device is intended to augment or replace an impaired function in an otherwise neurotypical individual rather than to rehabilitate a superimposed neurological impairment. This case study is limited in its ability to generalize the results observed here to other individuals with similar impairments, as it only documents the outcomes achieved by a single participant. However, the fact that this participant was able to learn to use the BCI system and demonstrate gains in behavioral measures shows that it is clearly possible for at least some individuals with preexisting disabilities or with atypical neurological characteristics prior to stroke to learn to control such devices and to potentially benefit from rehabilitative applications of these of therapies. This result is also consistent with prior work showing that no greater mental workload is incurred by individuals with physical disabilities when using BCI devices compared to healthy neurotypical control subjects performing the same tasks (Felton et al., 2012).

These results are promising both for the field of BCI technologies in stroke rehabilitation as well as for individuals with preexisting disabilities or neurological conditions that may benefit from them. It has been suggested that the minimal clinically important difference in ARAT improvement can range from as few as 6 points for chronic stroke patients (Van Der Lee et al., 2001) to 12 points for acute to subacute stroke patients with impairments of their dominant hand (Lang et al., 2008), and the participant's improvement in ARAT during the period in which BCI therapy was administered exceeded even a 12 point gain. In this case, these improvements in motor function also had a noticeable impact on the participant's ability to communicate, which may be reflected indirectly by his gains in SIS participation and more directly by the clinical observation that when communicating in the presence of the researchers the participant began using his impaired left hand to form ASL signs only after he began receiving BCI therapy.

The subject made notable gains in other measures assessed as well, showing concrete improvements in grip strength upon completion of BCI therapy as well as greater improvements in some subdomains of the SIS during the period during and after BCI therapy than during the pre-therapy observation period. Similarly, scores in each domain of the MAL increased during both the per-therapy observation period as well as during the period of BCI therapy administration, with the most dramatic increase being observed immediately surrounding the period of BCI therapy. However, the decline in scores for this measure at the final assessment in the absence of similar declines in the objective measures and in SIS scores may also reflect larger amounts of variability in MAL scores after the intervention, as the MAL has been shown to be most stable when assessing chronic stroke patients not undergoing an intervention (Van Der Lee et al., 2004), unlike the participant in this study. The improvements in MAL scores may also be reflecting, in part, improvements associated with the outside therapies administered or with study participation and interaction with study personnel, which were at a minimum during the 1 month period between the cessation of BCI therapy and the final assessment.

One major limitation of this study is the inability to clearly separate the effects of the interventional BCI therapy from effects of the additional occupational therapy, physical therapy, and botulinum toxin treatments that the participant received during the study period. Therefore, it is necessary to acknowledge that some or all of the functional gains described above may be attributed to spontaneous recovery, to the standard (i.e., non-BCI) therapies that the participant received during the study period, or to some combination thereof. However, although it is possible that none of the improvements described in this study were induced or facilitated by the BCI therapy, it seems unlikely that BCI therapy made no contribution to the gains observed specifically during the period during which BCI therapy was administered.

It is similarly not possible to determine what relative contributions various components of the BCI therapy, such as the neurofeedback training, the repeated attempted movement, or the FES, may have had to the recovery observed. However, the participant's BCI performance results suggest that he remained engaged in the neurofeedback task throughout the study period, so the gains documented during the therapy period do appear concurrent with regular neurofeedback training. While this study is unable to differentiate among the rehabilitative effects of interventional BCI therapy components and the effects of outside therapies and spontaneous recovery, it does serve as a demonstration for the potential of BCI therapy to serve as a component of an individualized rehabilitative regimen that may on the whole lead to gains in post-stroke motor recovery for individuals with preexisting disabilities.

It is also worth noting that the subject actually received more physical and occupational therapy during the observational control period of the study than during the period when BCI therapy was being administered and no outside therapies during the final weeks of BCI therapy and post-BCI therapy follow-up. Thus, the improved gains observed during and after BCI therapy occurred during a time period when additional therapies were either reduced or stopped altogether.

With regard to botulinum treatments, some studies have found botulinum toxin to improve upper extremity use and reduce disability in patients with upper extremity spasticity following stroke (Brashear et al., 2002; Pandyan et al., 2002; Rousseaux et al., 2002), but improvements in such studies are often highly variable. Some such gains are based on subjective self-report alone (Brashear et al., 2002), while others document improvements in objective measures of hand function in fewer than half the individuals receiving botulinum toxin treatment (Pandyan et al., 2002). One systematic review of neuromuscular blockade in upper extremity spasticity found that while tone may improve with botulinum toxin treatment, no clear functional improvements could not be convincingly demonstrated in the literature (Van Kuijk et al., 2002). Furthermore, it is important to note that by chance the participant's botulinum toxin schedule was similar between the pre-therapy and BCI therapy periods in that botulinum toxin was administered within 13 days of each baseline assessment (i.e., the first pre-therapy observational assessment and the assessment just before the initiation of BCI therapy). However, the most significant functional gains observed were seen only with BCI therapy administration during a time when spasticity measures either increased or remained similar to pre-therapy levels (Figure 7). Together, the similarity in botulinum toxin schedules and the absence of significant reductions in measured spasticity between the observational and BCI therapy periods suggest that the improvements documented with BCI therapy are unlikely to be attributable to the patient's botulinum toxin therapy.

While there is no way to definitively establish the cause of the increase in finger flexor spasticity observed near the end of the therapy period, one possible explanation for this finding is that finger flexors may have been strengthened during therapy when faced with increased resistance posed by the FES of the antagonizing extensor muscles. With the subject attempting repeated opening and closing of the hand and stimulus applied only to the extensor digitorum, repeated attempts to activate finger flexors during or just after extensor stimulation may have resulted in increases in strength and tone of the finger flexors. This increase in strength or tone of finger flexor muscles may also have contributed to the observed increases in the participant's ability to generate measurable grip strength near the end of the therapy period.

While most stroke survivors with motor impairments experience some functional recovery in the acute and subacute stroke periods either spontaneously or with traditional rehabilitative therapies, many reach a functional plateau within the first 6 months to 1 year after stroke and are left with motor impairments that can persist years. It has been suggested that the potential for further recovery remains in stroke survivors who have reached such a plateau and that newer therapies may facilitate this recovery where traditional therapies have ceased to produce measurable gains (Cramer, 2010). BCI therapy is one class of these newer therapies thought to help harness this potential for additional recovery. EEG-based BCI devices, such as the one employed in this study, detect the user's neural activity in the form of EEG signals and use these signals to provide real time feedback. These BCI systems have been coupled with various feedback modalities, including visual (Prasad et al., 2010), robotics (Buch et al., 2008; Broetz et al., 2010; Caria et al., 2011; Ramos-Murguialday et al., 2013; Varkuti et al., 2013), FES (Daly et al., 2009; Takahashi et al., 2012), and TDU (Wilson et al., 2012) by which the user can learn to modulate their brain activity.

In the context of motor rehabilitation therapy, this type of feedback is thought to strengthen central-peripheral connections and promote neuroplastic change by rewarding the production of consistent patterns of brain activation in conjunction with the subject's intent to move. Early studies of stroke survivors using such devices for rehabilitation have been promising, demonstrating functional gains (Buch et al., 2008; Daly et al., 2009; Broetz et al., 2010; Prasad et al., 2010; Caria et al., 2011; Shindo et al., 2011; Liu et al., 2012; Takahashi et al., 2012) as well as concurrent changes in brain activity and organization (Caria et al., 2011; Ramos-Murguialday et al., 2013; Varkuti et al., 2013). In this study, the changes in brain activation accompanying the participant's functional gains suggest that the same types of brain-behavior relationships documented in stroke survivors who receive BCI therapy may also play a role in motor recovery in stroke survivors with preexisting neurological conditions unrelated to their stroke.

With regard to the increased contralesional activity observed upon completion of the BCI therapy period (Figure 9B), this may be indicative of greater recruitment and coordination of contralesional brain areas during attempted finger tapping of the impaired left hand. While some studies examining changes in brain activation with BCI therapy found shifts in activity of the motor and premotor cortices toward the ipsilesional hemisphere to accompany motor gains (Caria et al., 2011; Ramos-Murguialday et al., 2013), others examining the role of the contralesional hemisphere in post-stroke recovery have shown that brain activation in the contralesional hemisphere and its functional connectivity to that of the ipsilesional hemisphere may contribute significantly to motor performance after stroke (Lotze et al., 2006; Carter et al., 2010). There have also been studies of other functional domains, such as language, where a compensatory shift in activation toward the intact hemisphere has been documented in response to infarction and hypoperfusion (Prabhakaran et al., 2007). As these changes were observed over the same time period in which significant functional motor gains were achieved, it is possible that this pattern may reflect neuroplastic processes related to the participant's motor recovery.

While the increased contralesional activity observed after BCI therapy cannot be directly attributed to the BCI therapy, it can be distinguished from brain changes observed with repeated scanning before and after the observational control period. The increased activity in the contralesional hemisphere with BCI therapy is noticeably different from the general decrease in ipsilesional activity observed over the course of the observational control period (Figure 9A). These different patterns of change suggest that the increases observed during the BCI therapy period are less likely to be due to a practice effect associated with repeated scans or due to effects associated with the outside physical therapy, occupational therapy, or botox treatments the participant received during both phases of the study period. Unfortunately, as this study documents such changes in only a single subject, it cannot be determined if the pattern of increased contralteral activations observed after BCI therapy is due to the nature of the BCI device used, some neurological characteristic of this subject that may or may not generalize to other individuals with preexisting sensorineural disabilities, or some interaction of the two. Future studies are needed to clarify whether the use of BCI devices is accompanied by differential changes in neural activity in individuals recovering from stroke and to determine what quantitative relationships may exist between such changes and increases in motor function.

The relationship between what parameters are implemented during BCI therapy and how these parameters may affect functional outcomes is another area in need of further investigation. In particular, overall BCI performance accuracy in this study was consistently above chance but not significantly greater than 70%, which is a commonly used threshold for establishing adequate control of a BCI system. Although this 70% was used as a threshold for increasing task difficulty, future participants may benefit from additional training either before additional modes of feedback are applied or before game play parameters are adjusted to make the task more difficult. The optimal balance between what performance accuracy should be demonstrated as an indication of adequate feedback vs. what level of difficulty should be implemented to minimize subject fatigue and/or boredom remains to be determined. Future studies are needed to better characterize this trade off in individuals with and without preexisting disabilities.

It will be necessary in the future to continue studying subpopulations of stroke survivors in order to better understand what, if any, differences there may be in the potential benefits of BCI therapy for those with preexisting disabilities or neurological conditions compared to the benefits that such therapies may offer individuals who were neurotypical prior to stroke. With nearly 10% of adults in the United States suffering from mood disorders such as depression (Kessler et al., 2005) and approximately 30% of adults in the United States aged 65 and older reporting a visual, hearing, or cognitive disability (Institute, 2013), it is important to ensure that advances in stroke rehabilitation can be made available to stroke survivors with preexisting disabilities or neurological conditions. This case study shows that a limitation such as deafness or a preexisting diagnosis such as depression may not be sufficient grounds upon which to deny such therapies to stroke survivors in need of rehabilitation, and further research will be needed to increase the availability of such therapies to stroke survivors with persistent motor impairments both in the presence or absence of preexisting neurological conditions.

## Author contributions

Brittany M. Young assisted in subject recruitment, data collection, data analysis, and writing. Zack Nigogosyan assisted with data collection and writing. Veena A. Nair assisted with subject recruitment, data collection, data analysis, and writing. Léo M. Walton assisted with data collection and writing. Jie Song assisted with subject recruitment and data collection. Mitchell E. Tyler provided TDU hardware and expertise. Dorothy F. Edwards assisted with study design and data analysis. Kristin Caldera assisted with subject recruitment. Justin A. Sattin assisted with study design. Justin C. Williams is one of two lead PI's on this project and supervised the technical and engineering aspects of the work. Vivek Prabhakaran is one of two lead PI's on this project and supervised the neuroimaging and neuroscience aspects of this work.

### Conflict of interest statement

There is one patent pending on the closed-loop neurofeedback BCI-FES-TDU device, filed jointly by the two senior PI's, Justin C. Williams and Vivek Prabhakaran, who oversaw this work (patent application no. 12/715,090). Otherwise, this research was conducted without significant commercial or financial relationships.

## Supplementary material

Video S1 **Video of the participant's attempted hand movements during the open-loop screening task showing attempted repeated hand opening and closing for each hand as well as rest on the first day of interventional BCI therapy**. Words in the bottom left corner of the screen correspond to the stimuli shown to the participant over the course of the video period. Stimuli were presented in random order. Video was taken with the camera positioned above the table facing the participant such that the participant's left hand is shown on the right side of the screen.
